# The X-Linked *TLR7* rs179008 T Allele Is Associated with an Increased Risk of Severe Multisystem Inflammatory Syndrome in Children/Kawasaki-like Syndrome in SARS-CoV-2-Infected Boys

**DOI:** 10.3390/ijms26178491

**Published:** 2025-09-01

**Authors:** Adriana de Souza Andrade, Aline Almeida Bentes, Lilian Martins Diniz, Silvia Hees Carvalho, Erna Geessien Kroon, Marco Antonio Campos

**Affiliations:** 1 Instituto René Rachou, Fiocruz Minas, Belo Horizonte 30190-009, MG, Brazil; andradeadriana.ds@gmail.com (A.d.S.A.); silviahees@outlook.com (S.H.C.); 2 Departamento de Pediatria, Universidade Federal de Minas Gerais, Belo Horizonte 30130-110, MG, Brazil; alinebentes2000@gmail.com (A.A.B.); lilianmodiniz@gmail.com (L.M.D.); 3 Hospital Infantil João Paulo II, Minas Gerais, Belo Horizonte 30130-110, MG, Brazil; 4 Laboratório de Vírus, Departamento de Microbiologia, Universidade Federal de Minas Gerais, Belo Horizonte 31270-201, MG, Brazil; ernagkroon@gmail.com

**Keywords:** SARS-CoV-2, severe multisystem inflammatory syndrome in children, Kawasaki-like syndrome, toll like receptor, *TLR7*, rs179008 T

## Abstract

The X-linked *TLR7* rs179008 T allele has been associated with altered antiviral immunity. Given their shared inflammatory pathways and higher pediatric mortality rates in Brazil during the pandemic, we investigated their association with multisystem inflammatory syndrome in children (MIS-C) together with Kawasaki disease (KS) following SARS-CoV-2 infection. A cross-sectional study (2021–2022) analyzed 73 hospitalized children (<13 years) with confirmed COVID-19. Genotyping for *TLR7* rs179008, *TLR8* (rs3764879, rs2407992), and *TLR3* rs3775291 was performed via PCR and Sanger sequencing. MIS-C/KS cases were identified using CDC criteria, with severity classified by the need for ICU care. Statistical analysis included Fisher’s exact test and relative risk (RR) calculations. Hemizygous boys carrying the *TLR7* T allele had a 1.87-fold higher risk of MIS-C/KS (*p* = 0.007) and a 1.75-fold increased risk of severe or critical outcomes. The T allele frequency was 2.6× higher in MIS-C/KS cases versus other COVID-19 presentations. All fatalities occurred in boys (3/8 MIS-C cases) with one T-allele carrier. No associations were found for *TLR8* or *TLR3* variants. The *TLR7* rs179008 T allele is a potential genetic risk factor for severe post-COVID-19 inflammatory syndromes in boys, likely due to impaired immune signaling. These findings highlight its utility as a biomarker for risk stratification in pediatric populations.

## 1. Introduction

During the COVID-19 pandemic, 4.4 million people died worldwide, and only 0.4% were children and adolescents [1,2]. In 2020, Brazil recorded 23 deaths per 1,000,000 children, and this rate increased to 32 deaths per 1,000,000 in 2021 [1]. By mid-2022, over 1500 COVID-19-related deaths had been reported among children aged 0 to 11 years, making the disease one of the leading causes of child mortality in the country [3]. While most children infected with SARS-CoV-2 experience asymptomatic or mild illness, severe disease can develop, including acute respiratory distress syndrome and necrotizing pneumonia as key pulmonary manifestations, along with various forms of cardiovascular and neurological involvement [4].

Among the possible complications, Multisystem Inflammatory Syndrome in Children (MIS-C) and Kawasaki Syndrome (KS) pose significant risks, including mortality. MIS-C typically manifests 2 to 6 weeks after infection with SARS-CoV-2 and is characterized by persistent fever, involvement of multiple organ systems, and elevated markers of systemic inflammation More than 50% of affected children require Intensive Care Unit (ICU) admission, and cases occur even in previously healthy individuals [5,6].

KS is a febrile pediatric systemic vasculitis of unknown etiology, predominantly affecting children under five years of age, with higher prevalence in males. It causes inflammation of medium-sized arteries and may lead to severe cardiovascular complications, particularly coronary artery aneurysms, which develop in about 25% of untreated cases [6]. Despite decades of research, its triggers, immunopathological mechanisms, and long-term cardiovascular consequences remain unclear [7].

Early in the COVID-19 pandemic, MIS-C was described as “Kawasaki-like”, a pediatric febrile vasculitis capable of causing coronary artery aneurysms [5]. Although distinct, MIS-C and KS share overlapping features such as conjunctival injection, mucosal inflammation, and peripheral edema, suggesting a potential common inflammatory pathway [5,6,7,8]. This considerable overlap in systemic inflammation and mucocutaneous manifestations highlights their similarities and supports the hypothesis of partially shared pathogenic mechanisms [5,8,9,10].

The pathogen-host interaction and the innate immune response are key determinants of disease prognosis in both diseases, particularly through the type I interferon and inflammasome pathways. MIS-C and KS highlight the potential dangers of an exaggerated immune response, which can lead to widespread inflammation affecting multiple organs [4,10,11]. Given the potentially shared inflammatory pathways and the initial difficulty distinguishing between the two conditions during the early stages of the pandemic, this study will investigate the impact of toll like receptor gene (*TLR*) mutations in both syndromes as potentially related inflammatory disorders.

Type I interferon (IFN-I) and inflammasome pathways are pivotal to the immune response against infections, with TLRs playing a key role in their activation. TLRs are a class of receptors in the innate immune system that recognize pathogen-associated molecular patterns (PAMPs) and initiate downstream immune responses. Both KS and MIS-C are believed to result from an aberrant immune response to unidentified infectious or environmental stimuli [12,13]. Therefore, could an inadequate immune response to SARS-CoV-2 infection, caused by a lack of TLR signaling, be associated with the development of MIS-C and KS? *TLR7* and *TLR8* recognize single-stranded RNA (ssRNA), while *TLR3* recognizes double-stranded RNA (dsRNA). These ligands are the majority of viral origin, highlighting the critical role of these receptors in antiviral defense, particularly against RNA viruses such as SARS-CoV-2 [14,15]. Specifically, *TLR7* senses viral RNA and triggers the production of IFN-I along with other cytokines, thereby limiting viral replication and enhancing the host’s antiviral response [16,17,18].

Single-nucleotide polymorphisms (SNPs) can give rise to *TLR7* mutations, resulting in a loss of receptor function, which may impair downstream signaling. The *TLR7* SNP rs179008 (A > T) has been associated with increased severity in infectious diseases [19,20]. In a previous study conducted by our group, we observed a higher prevalence of the rs179008 mutation in severe and critical COVID-19 cases among 73 children hospitalized in a hospital in Belo Horizonte, Brazil [21].

In addition to *TLR7* SNPs, variants in the *TRL8* and *TLR3* genes were also described. The SNP rs3764879 (−129 G > C), located in the promoter region of the *TLR8* gene, has been associated with altered transcriptional activity and reduced mRNA stability, affecting protein production and monocyte cytokine responses [22]. Another *TLR8* variant, rs2407992 (G > C) in a coding region, has been evaluated in silico. The *TLR3* SNP rs3775291 (C > T) is a non-synonymous mutation resulting in a Leu412Phe substitution; this variant impairs dsRNA binding and destabilizes the ectodomain, thereby attenuating receptor signaling. *TLR7* and *TLR8* are located on the X chromosome, while *TLR3* is on chromosome 4 [18].

We hypothesize that these SNPs may influence progression to KS and MIS-C following COVID-19; therefore, this study aims to investigate the prevalence of these *TLR* variants in children who developed MIS-C and KS after contracting COVID-19.

## 2. Results

### 2.1. Pediatric Study Profile and Disease Severity

**Figure 1:**
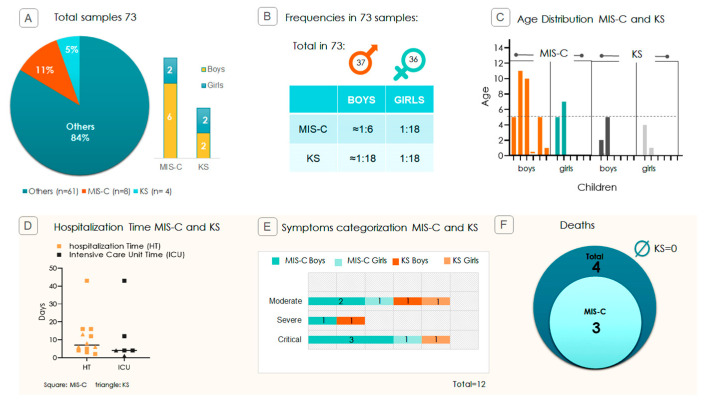
Pediatric study profile and disease severity. (**A**) Proportion (in percentage) of MIS-C and KS cases among the 73 SARS-CoV-2-infected children. The panel displays absolute counts and percentages stratified by sex (boys vs. girls). (**B**) Incidence of MIS-C and KS by sex, calculated from 37 boys and 36 girls, illustrating ~3:1 male bias in MIS-C cases. (**C**) Age distribution of affected children; each bar on the X-axis represents one child, and the Y-axis corresponds to age. The trendline indicates that most children are aged ≤ 5 years. (**D**) Hospitalization metrics: total hospitalization time (HT) and Intensive Care Unit time (ICU) duration in days. Squares represent MIS-C cases, and triangles represent KS cases. (**E**) Clinical severity distribution among MIS-C and KS cases categorized as moderate, severe, or critical. (**F**) Diagram showing that out of the 4 deaths in the 73 samples, 3 were associated with the progression of symptoms to MIS-C and none associated with KS (Ø = no deaths).

From a previous study involving 73 children with various symptoms of SARS-CoV-2 infection [21], we identified 8 cases (11%) who progressed to MIS-C, including 6 boys and 2 girls, and 4 cases who progressed to KS (5%), 2 boys and 2 girls (Figure 1A). Among MIS-C cases, boys were approximately three times more affected, with one case per 6 boys and one case per 18 girls (Figure 1B). Most cases of both syndromes occurred in children under 5 years old (Figure 1C). The hospitalization rate in the ICU (Intensive Care Unit) for both MIS-C and KS was 50% (4 of 8 and 2 of 4 cases, respectively). The median hospitalization duration was 8.5 days, and the median ICU stay was 4 days among those requiring intensive care. Notably, one child experienced significantly more extended hospitalization and ICU stay (Figure 1D). Cases were classified as moderate, severe, or critical, with most falling into the severe and critical groups (Figure 1E). Of the four fatalities among the 73 children, three were associated with progression to MIS-C (Figure 1F).

**Figure 2:**
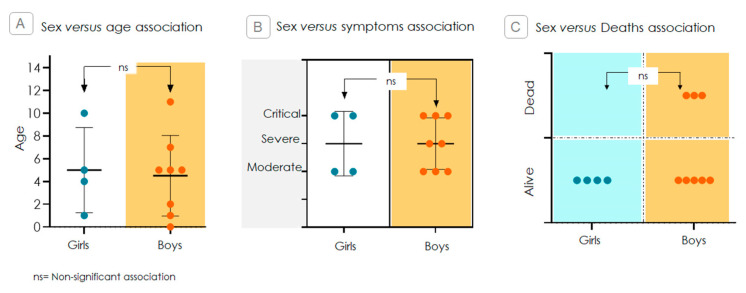
Sex-specific comparison of clinical parameters in children with MIS-C/KS following SARS-CoV-2 infection. Contingency analyses using Fisher’s exact test were performed to assess associations across age, symptom severity, and mortality. Data are presented as medians ± standard deviations. Panels (**A**,**B**) show non-significant differences (ns) in age distribution and clinical severity by sex. Panel (**C**) indicates no statistically significant difference in mortality between boys and girls (*p* = 0.4909), although all deaths occurred in male patients.

The distribution of various parameters, including age, symptom severity, and mortality outcome, was analyzed by the sex of the children. No statistically significant differences were observed in the age or severity distribution between boys and girls (Figure 2A,B). Similarly, mortality distribution did not show significant sex-based differences. However, it is noteworthy that all recorded deaths occurred exclusively in boys (Figure 2C).

### 2.2. Association Between TRLs and the Risk of Progression to MIS-C/KS

**Figure 3:**
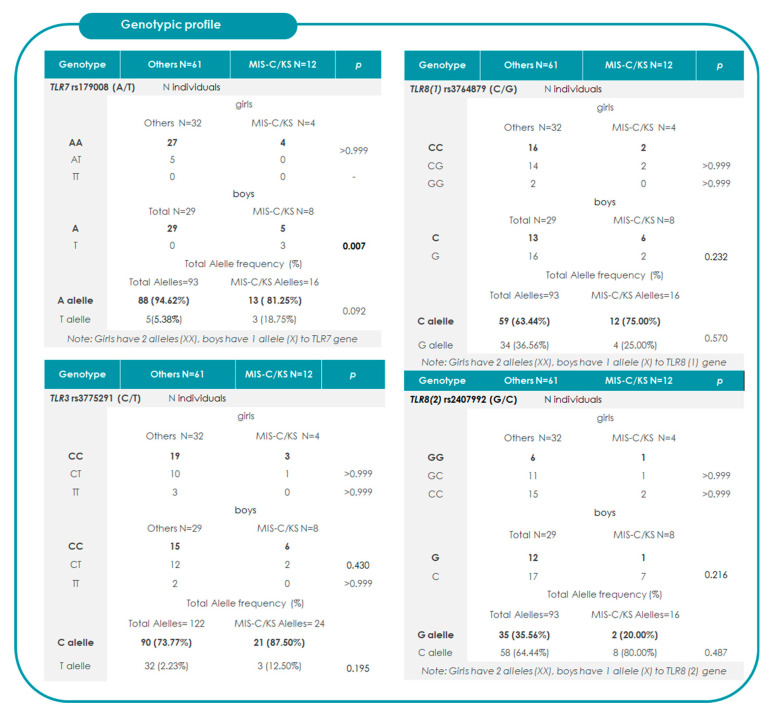
Genotypic profile of *TLR7*, *TLR3, TLR8* (1), and *TLR8* (2) variants in children with MIS-C/KS compared to other SARS-CoV-2 symptoms. Allelic and genotypic frequencies are presented for *TLR7* rs179008 (A/T), *TLR3* rs3775291 (C/T), *TLR8* (1) rs3764879 (C/G), and *TLR8* (2) rs2407992 (G/C) loci. The data include the number of individuals with other symptoms, excluding MIS-C/KS, from our previous SARS-CoV-2 study (N = 61) [21], as well as the present study, which focused on children with MIS-C/KS (N = 12), including both male and female children. *TLR7* and *TLR8* are on the X chromosome, and *TLR3* is on chromosome 4. It is important to note that for *TLR7* and *TLR8*, girls have two X chromosomes (XX) and could, therefore, exhibit AA, AT, or TT genotypic profiles for *TLR7* rs179008. In contrast, boys have only one X chromosome, so their genotypic profile can be either A or T for *TLR7* rs179008. Boys are hemizygous (A or T); girls could be homozygous (AA, TT) or heterozygous (AT). Total allele frequency refers to the number of alleles, considering the allelic differences between boys and girls. Percentages of allele frequencies are shown in parentheses. The *p*-value was calculated from the contingency table using Fisher’s exact test. The reference for analyzing the impact of the mutations is the comparison with the non-mutated genotype, highlighted in bold in the table. *p*-values < 0.05 are significant.

When analyzing the *TLR7* rs179008 (T) allele within our SARS-CoV-2 study, which includes the MIS-C/KS subgroup, we observed that all girls carrying the variant were heterozygous (AT genotype); no TT homozygotes were identified. In boys who are hemizygous due to having only one X chromosome, the presence of the T allele functionally equates to homozygosity. Due to this fact, the analyses of the association between MIS-C/KS and the *TLR7* rs179008 (T) allele in girls or in boys applies only to boys, since there were no homozygous in girls in this study. The values of the identified genotypes are detailed in Figure 3. Contingency analysis revealed that in female heterozygotes, the T allele heterozygote was not associated with progression to MIS-C. However, in boys, the presence of a single T allele showed a significant correlation with progression to MIS-C/KS (*p* = 0.007). No significant associations were observed for *TLR8* (1 and 2) or *TLR3* gene variants.

### 2.3. Relative-Risk Analysis for the Association of *TLR7* rs179008 in Relation to Progression to MIS-C/KS and Clinical Severity

**Figure 4:**
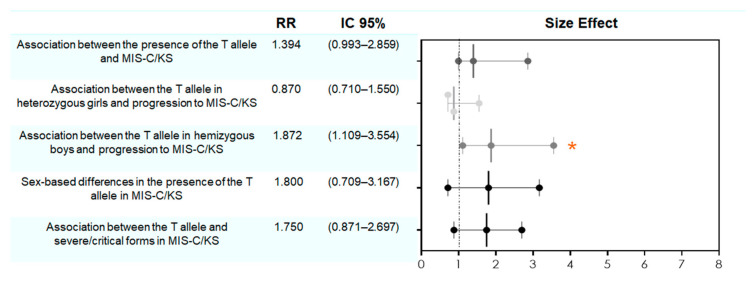
Relative-risk analysis of the *TLR7* rs179008 T allele in relation to progression to MIS-C/KS and clinical severity. *TRL7* rs179008, boys are hemizygous (A or T); girls could be homozygous (AA, TT) or heterozygous (AT), but they were only homozygous in this study. Forest plot displaying relative risks (RR) and 95% confidence intervals (CI) for the presence of the T allele across multiple clinical profiles. Contingency tables were generated, with RR and CI calculated using the Koopman asymptotic score, and statistical significance assessed via Fisher’s exact test. Points to the right of the vertical “no effect” line (RR = 1) indicate a positive association; points to the left indicate a negative association, and intervals crossing the line denote non-significant relationships. The orange asterisk indicates statistical significance.

Since no significant associations were observed for *TLR8* (1 and 2) or *TLR3* gene variants, and because no significant associations were observed for girls, the subsequent analyses were focused exclusively on *TLR7* in males. Relative-risk and confidence interval analyses were performed for various parameters concerning *TLR7*, as depicted in Figure 4. We identified a statistically significant association (*p* < 0.05) between the T allele in hemizygous boys and progression to MIS-C/KS, indicating a 1.87-fold increased risk of these conditions in children carrying this variant. Sex-specific associations showed elevated relative risks for the T allele in boys compared to girls (RR = 1.80) and severe/critical MIS-C/KS cases (RR = 1.75), although these did not reach statistical significance (*p* > 0.05).

**Figure 5:**
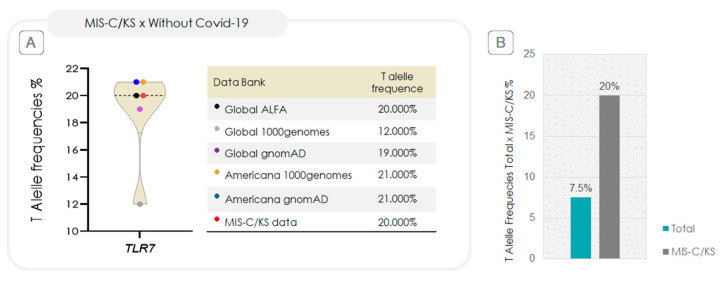
Comparison of *TLR7* rs179008 T allele frequencies between MIS-C/KS cases and reference populations. (**A**) Violin blot illustrating T allele frequency among children with MIS-C/KS, compared to five public population databases representing individuals without COVID-19. The dashed line represents the median. Frequencies in the MIS-C/KS group did not differ significantly from those in the reference databases. Minimum–maximum vertical lines indicate the full range, and the shape reflects the distribution density. (**B**) Bar graph comparing T allele frequency between the pediatric groups from our study: total of children with SARS-CoV-2 infection (N = 73, blue) [21] and those who developed MIS-C/KS (N = 12, gray).

The genotypic frequency of the T allele in the MIS-C/KS group closely matches that reported in major population databases, as shown in Figure 5A. However, when compared to the overall samples of 73 SARS-CoV-2–infected children [21], the T allele frequency in the MIS-C/KS subset is approximately 2.6-fold higher, as illustrated in Figure 5B.

### 2.4. Is the TLR7 rs179008 T Allele Associated with Increased Mortality?

**Figure 6:**
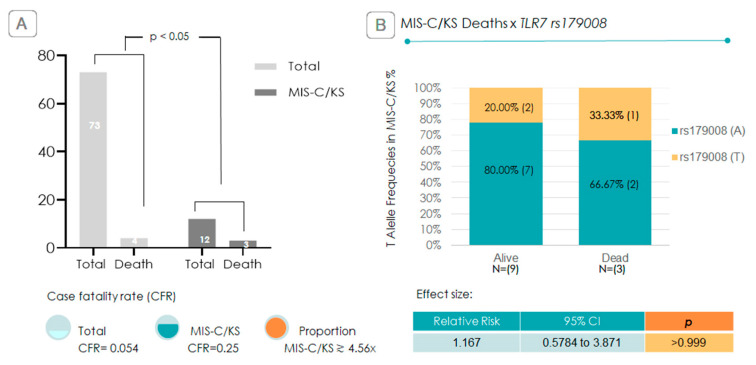
Lethality comparison and T allele association in MIS-C/KS cases. (**A**) Bar chart comparing case fatality rates (CFR) among 73 children hospitalized with COVID-19 versus the 12 children who developed MIS-C/KS. The fatality rate in the MIS-C/KS subgroup is approximately 4.56-fold higher than the total of 73 COVID-19 hospitalized children (*p* < 0.05). (**B**) Relative-risk analysis of the *TLR7* rs179008 T allele in MIS-C/KS cases, stratified by survival. Below is the description of the relative risk, confidence interval (95%), and *p*-value for the T genotype appearing in death cases.

Among the 73 cases, there were 4 deaths overall, 3 of which followed progression to MIS-C/KS. The overall case fatality rate was 5% (0.05), whereas within the MIS-C/KS subgroup, it increased to 25% (0.25), representing a fivefold rise. Specifically for MIS-C alone, the fatality rate was 37.5%. Within the MIS-C/KS group, all three children who died were male, and one of them carried the *TLR7* rs179008 T allele. Among the nine survivors, two were also T allele carriers (Figure 6). Although this sample size precludes statistical significance, a trend emerges: the *TLR7* rs179008 T allele was present in one of the three fatal cases of MIS-C/KS in a population where the allele frequency was relatively low (7.5%—Figure 5). This corresponds to a 1.17-fold increased relative risk of death associated with the T allele in MIS-C/KS cases (Figure 6), although the association did not reach statistical significance (*p* > 0.05) (Figure 6).

## 3. Discussion

The association between *TLR7* rs179008 T and the development of MIS-C/KS suggests its potential role in influencing the severity of immune responses to SARS-CoV-2 in pediatric patients. This finding could provide valuable insights into the genetic and immunological factors that contribute to severe COVID-19 outcomes in children. Identifying these associations is crucial, as it might reveal biomarkers that can predict susceptibility to MIS-C/KS. This knowledge could lead to earlier detection, more targeted treatment strategies, and personalized healthcare interventions for those at highest risk.

In our study, 10.95% (8/73) of hospitalized children developed MIS-C, closely matching 9% (6/66) incidence reported by Pereira et al. (2020) [23]. Additionally, KS occurred in 5.5% (4/73) of the children, a rate nearly identical to the 5.1% reported in a multi-country COVID-related cohort by Molloy et al. (2022) [24]. Among the children with MIS-C, 50% were admitted to the ICU (Figure 1), which is somewhat lower but comparable to the 73.3% ICU admission rate found in a multicenter study from São Paulo, Brazil [7]. In contrast, KS in our study also had a 50% ICU admission rate, significantly higher than the 19% reported by Hufnagelet al. (2023) [25]. Finally, we observed that boys were three times more likely than girls to develop MIS-C (6 boys versus 2 girls). This finding is consistent with a Brazilian multicenter prospective study in which 70% of MIS-C patients were male [26].

Although epidemiological and immunological features often distinguish MIS-C and Kawasaki disease (KD), some authors argue they represent variations within the same clinical spectrum. Both conditions share systemic hyperinflammation, vasculopathy, cytokine storm, and responsiveness to intravenous immunoglobulin. They also exhibit overlapping biomarkers, including elevated levels of C-reactive protein (CRP), ferritin, D-dimer, erythrocyte sedimentation rate (ESR), fibrinogen, pro-B-type natriuretic peptide (pro-BNP), and various cytokines like IL-1β, IL-6, IL-10, tumor necrosis factor (TNF), interferon-gamma (IFN-γ), and the IL-15/IL-15RA axis. Additional similarities include the formation of neutrophil extracellular traps (NETs), activated monocytes, and lymphopenia, which suggest convergent immune mechanisms. From this perspective, KD could be viewed as a heterogeneous syndrome triggered by multiple pathogens in genetically susceptible hosts. Meanwhile, MIS-C might represent an intensified immune response to SARS-CoV-2, which explains its distinctive features such as gastrointestinal and neurological involvement, myocarditis, and later age at onset [8,27].

Due to their overlapping symptomatology, pathogenesis, and inflammatory pathways, we combined MIS-C and KS [5,8,9,10] for analysis against other COVID-related symptoms in our SARS-CoV-2 group of 73 children from our previous study [21]. In our previous work, we observed increased frequencies of *TLR3*, *TLR7*, and *TLR8* variants among severe and critical COVID-19 cases. We therefore assessed whether these same SNPs might also be associated with MIS-C and KS development in this expanded analysis.

Initially, we analyzed clinical variables, including sex, age, symptoms, and lethality, and found no significant differences that affected downstream analyses (Figure 2). Next, we compared SNP profiles relative to the ancestral allele, examining the frequencies of heterozygous and hemizygous variants among children with other COVID-19 symptoms and those who developed MIS-C/KS. As shown in Figure 3, only the rs179008 T allele in *TLR7* gene, under monoallelic expression, demonstrated a statistically significant association, a pattern noted exclusively in boys. Based on this finding, we conducted a focused analysis to explore its biological relevance and implications.

Association analyses revealed a statistically significant link between *TLR7* rs179008 T allele and progression to MIS-C/KS in boys with a relative risk (RR) of 1.872. In other words, boys carrying the T allele (hemizygous) had an 87.2% higher risk of developing MIS-C/KS compared to the other 61 hospitalized children with COVID-19 (Figure 3 and Figure 4). No such correlation was observed in girls, likely because T-allele carriers in females (in this study) were heterozygous (AT genotype). One plausible hypothesis is that allelic expression of the T allele in boys significantly impairs functional *TLR7* production. At the same time, heterozygous girls retain sufficient receptor expression from the normal allele, preserving an adequate immune response [28,29]. Studies in males with deleterious *TLR7* variants support this sex-based vulnerability to COVID-19, suggesting that the *TLR7* gene-dose effect renders males more susceptible to severe outcomes [29,30,31,32]. Since no females homozygous for the TT genotype were present in our sample, we cannot determine whether biallelic T-allele expression in girls would confer a similar risk profile to that seen in hemizygous boys, meaning they have only one copy of each gene on that chromosome. Future studies with larger and more diverse cohorts are needed to explore this possibility.

In our study, MIS-C/KS cases were more frequent among boys, and all four recorded deaths occurred in male patients. We also observed a 1.75-fold increase in relative risk of developing severe or critical MIS-C/KS in children with monoallelic expression (profile found only in boys) of the *TLR7* rs179008 T allele, compared to other children in MIS-C/KS group carrying the wild-type allele (Figure 4). This suggests that hemizygous T-allele expression in males is linked to more severe clinical outcomes.

The frequency of the *TLR7* rs179008 T allele in our study closely aligns with data from large polymorphism databases (Figure 5A). Notably, in our study population, the T allele was approximately 2.6 times more frequent in the MIS-C/KS subgroup (Figure 5B). This finding, along with the statistically significant association between the *TLR7* rs179008 T allele (in hemizygous boys) and MIS-C/KS, as well as the increased relative risk for severe and critical presentations in affected MIS-C/KS children, supports the hypothesis that these genetic factors may contribute to disease severity.

*TLR7*, which is found in endosomal compartments, plays a crucial role in recognizing viral single-stranded RNA. It signals through the MyD88-dependent pathway, leading to the production of type I interferons (IFN-α/β) and proinflammatory cytokines, via NF-κB [18,33]. This process is essential for establishing both innate and adaptive antiviral defenses. When *TLR7* function is impaired, the early detection of viruses is compromised, resulting in delayed interferon responses and prolonged viral replication [34]. In children, mucosal surfaces typically produce type I interferons more effectively, which are vital for local protection against SARS-CoV-2 [35]. However, reduced *TLR7* activity can weaken this response, allowing the virus to persist longer and increasing the risk of severe inflammatory conditions such as MIS-C and KD. Following this ineffective antiviral phase, a compensatory but dysregulated activation of inflammatory pathways may occur, including exacerbated nuclear translocation of NF-κB, and consequent higher production of IL-6 and TNF-α. This can lead to the cytokine storm observed in these syndromes. Additionally, prolonged viral presence and immune dysregulation can result in the accumulation of immune complexes and increased activation of polyclonal B cells, further intensifying systemic inflammation. In summary, *TLR7* deficiency may lead to an inadequate early antiviral response followed by uncontrolled immune activation, which likely contributes to the immunopathogenesis of MIS-C and KD [36].

Regarding CFR, it is well established that progression to MIS-C significantly increases the risk of death, as demonstrated in studies by Pereira (2020) [23], Feldstein et al. (2020) [37], and de Farias et al. (2024) [38]. De Farias et al. (2024) [38] found a CFR of 22.5% in a study conducted in the northern region of Brazil during a similar period. In our study, we found CFR for combined MIS-C/KS of 25%, which is 4.6-fold higher than the overall CFR among SARS-CoV-2-infected children. For MIS-C alone, the CFR reached 37.5%, and notably, all deaths occurred exclusively in the MIS-C group and none in children with KS.

Given the limited sample size, statistical significance testing tends to be more conservative; consequently, achieving *p*-values < 0.05 in Fisher’s exact test becomes more challenging, even in the presence of potentially meaningful trends. Nonetheless, our findings support a potential association between the rs179008 T polymorphism and increased susceptibility to MIS-C/KS. If validated in large cohorts, this variant may serve as a predictive biomarker for severe pediatric COVID-19 outcomes and guide personalized clinical interventions, with important implications for public health and disease management. Nevertheless, the fact that statistically significant associations emerged despite this constraint suggests that the signal is robust and biologically relevant. Furthermore, even those results that did not reach statistical significance showed patterns that indicate a possible association, which could become more evident in larger studies.

## 4. Materials and Methods

### 4.1. Ethical Approval and Written Consent

The ethical approval for this study was obtained from the Instituto René Rachou Ethical Committee, Fiocruz (CAAE 37207920.6.0000.5091), and the Fundação Hospitalar do Estado de Minas Gerais (FHEMIG, CAAE 37207920.6.3001.5119). Written consent was obtained from the patient’s legal guardian.

### 4.2. Design and Sampling

This cross-sectional study, conducted between 2021 and 2022, focused on children under 13 years of age who were hospitalized at Hospital Infantil João Paulo II (FHEMIG) during the COVID-19 pandemic. Clinical data were collected for 73 children, and blood samples were obtained according to defined inclusion and exclusion criteria. Eligible participants were children hospitalized with signs of infection and a laboratory-confirmed diagnosis of COVID-19, determined by PCR, antigen testing, or IgM/IgG antibody detection. Participation required parental consent via a signed informed consent form and, when applicable, assent from the child through a signed assent form. Exclusion criteria included a history of immunosuppressive therapy or concurrent infections during an immunosuppressed state. These collected samples were processed and stored in the biobank of the Laboratório de Imunologia de Doenças Virais at Instituto René Rachou, Fiocruz, Brazil. Sequencing analyses were performed to assess mutations in the *TLR7* SNP rs179008, *TLR8* [rs3764879 (1) and rs2407992 (2)], and *TLR3* (rs3775291). The procedure involved DNA extraction, PCR amplification, electrophoresis, and purification of amplified products.

### 4.3. Identification and Severity Classification

Cases of MIS-C among the 73 pediatric patients were identified by the medical team based on the criteria established by the Centers for Disease Control (CDC) [39]. Severity was classified according to clinical presentation, adapted from The PODIUM Consensus Conference [40] evidence-informed criteria for pediatric organ dysfunction. For patients diagnosed with MIS-C or KS, severity was categorized into three levels: moderate for those who did not require ICU admission, severe for those who required ICU care, and critical for cases that necessitated vasopressors, inotropes, or mechanical ventilation. This stratification enabled a rigorous evaluation of case severity, facilitated comparative analysis, and enhanced understanding of the varied clinical profiles observed in the study [21].

### 4.4. DNA Extraction, Amplification, and Sequencing

Genomic DNA was extracted using the DNeasy Blood and Tissue Kit (Qiagen, Germany), following the manufacturer’s instructions. PCR amplification targeted the following SNPs: *TLR8* (1)—rs3764879, *TLR8* (2)—rs2407992, *TLR7*—rs179008, and *TLR3*—rs3775291, using primers and cycling conditions as described in Andrade et al., 2024 [21]. Sequencing was performed using the Sanger method [41]. Reactions were run on an ABI 3730xL Genetic Analyzer (Applied Biosystems, Waltham, MA, USA) at the Instituto René Rachou. Sequence reads were analyzed using SeqTrace software 0.9.0 [42], aligning forward and reverse reads, conducting quality assessments, and identifying mutations.

### 4.5. Sequencing and Statistical Analysis

Sequencing of *TLR7* rs179008, *TLR8* [rs3764879 (1) and rs2407992 (2)], and *TLR3* (rs3775291) was performed using SeqTrace [42], with quality checks and repeat analyses as necessary. For *TLR7*, males were hemizygous, while females could be either homozygous or heterozygous [43]. A total of 73 pediatric samples (<13 years old) were analyzed for these SNPs, including 8 MIS-C and 4 Kawasaki syndrome (KS) cases, which were compared to 61 pediatric COVID-19 cases with varying severity. Mutation data were evaluated using contingency tables, Fisher’s exact test (*p* < 0.05), relative risk analysis, and visualizations generated in GraphPad Prism 8 (Boston, MS, USA)

## 5. Conclusions

The *TLR7* rs179008 T allele is a potential genetic risk factor for severe post-COVID-19 inflammatory syndromes in boys, likely due to impaired immune signaling. These findings highlight its utility as a biomarker for risk stratification in pediatric populations.

## Data Availability

The original contributions presented in this study are included in the article. Further inquiries can be directed to the corresponding author.

## Abbreviations

The following abbreviations are used in this manuscript:

HT	Hospitalization time
ICU	Intensive care units
IFN-I	Type I interferon
KS	Kawasaki disease
MIS-C	Multisystem inflammatory syndrome in children
PAMP	Pathogen-associated molecular patterns
RR	Relative risk
SNP	Single-nucleotide polymorphisms
TLR, *TLR*	Tolllike receptor, gene *TLR*
